# Expanding the Spectrum of Adenoviral Vectors for Cancer Therapy

**DOI:** 10.3390/cancers12051139

**Published:** 2020-05-02

**Authors:** Jian Gao, Wenli Zhang, Anja Ehrhardt

**Affiliations:** Institute for Virology and Microbiology, Center for Biomedical Education and Research (ZBAF), Department of Human Medicine, Faculty of Health, Witten/Herdecke University, 58453 Witten, Germany; jian.gao@uni-wh.de (J.G.); wenli.zhang@uni-wh.de (W.Z.)

**Keywords:** adenovirus, cancer, adenoviral vectors, oncolytic adenovirus, delivery, immunotherapy, chemotherapy, radiotherapy, adenovirus library

## Abstract

Adenoviral vectors (AdVs) have attracted much attention in the fields of vaccine development and treatment for diseases such as genetic disorders and cancer. In this review, we discuss the utility of AdVs in cancer therapies. In recent years, AdVs were modified as oncolytic AdVs (OAs) that possess the characteristics of cancer cell-specific replication and killing. Different carriers such as diverse cells and extracellular vesicles are being explored for delivering OAs into cancer sites after systemic administration. In addition, there are also various strategies to improve cancer-specific replication of OAs, mainly through modifying the early region 1 (E1) of the virus genome. It has been documented that oncolytic viruses (OVs) function through stimulating the immune system, resulting in the inhibition of cancer progression and, in combination with classical immune modulators, the anti-cancer effect of OAs can be even further enforced. To enhance the cancer treatment efficacy, OAs are also combined with other standard treatments, including surgery, chemotherapy and radiotherapy. Adenovirus type 5 (Ad5) has mainly been explored to develop vectors for cancer treatment with different modulations. Only a limited number of the more than 100 identified AdV types were converted into OAs and, therefore, the construction of an adenovirus library for the screening of potential novel OA candidates is essential. Here, we provide a state-of-the-art overview of currently performed and completed clinic trials with OAs and an adenovirus library, providing novel possibilities for developing innovative adenoviral vectors for cancer treatment.

## 1. Introduction

Adenoviruses (Ads) belong to the *Adenoviridae* family and represent the largest known group of non-enveloped viruses. The virion is a medium-sized particle (90–100 nm) and contains a double-stranded DNA genome encapsidated in an icosahedral capsid. The capsid is mainly composed of hexon-, penton-, and fiber proteins, of which the latter can be divided into fiber knob and shaft. The virus is composed of around one million amino acid residues and weighs around 150 MDa [1]. Ads are associated with infections of various organs and can be observed in immunocompromised patients. To date, 103 human Ad types (Ad1 to Ad103) have been identified in HAdV Working Group [2] and classified into seven species (A to G) based on hemagglutination properties, oncogenicity in rodents, DNA homology, and genome organization [3,4]. An overview of all identified human Ad types and their specific characteristics, including receptor usage and tropism, are provided in Table 1. Human Ads infect a broad variety of cell types, including respiratory cells, renal cells, ocular cells, hepatic cells and gastrointestinal cells. During the infection process, Ads first contact the cell surface receptors such as coxsackievirus- and adenovirus receptor (CAR), CD46, CD80/86, desmoglein 2 (DSG2) and heparan sulfate proteoplycans (HSPG) [3,4,5,6]. Subsequently, the viruses enter cells and replicate in host cells.

As stated in the Journal of Gene Medicine [7], recombinant Ads are the most widely used viral vectors for gene therapy, accounting for 18.6% of vectors used in gene therapy clinical trials. Infection into both quiescent and dividing cells, a large cargo capacity of up to 37 kb and the transduction of a broad variety of cell types render AdVs as the most widely used vector system. Currently, AdVs show potential in different fields, including gene therapy, vaccine trials and cancer treatments as oncolytic viruses (OVs) [8,9].

The first oncolytic adenovirus (OA) Onyx-015 (*dl*1520) for pancreatic cancer was introduced to clinical trials in 2001 [10]. As research progressed, Onyx-015 demonstrated feasibility and safety but poor efficacy. The findings from Onyx-015 indicated the potential of AdVs in cancer treatments and inspired more research to optimize and improve them.

According to the documented research, the problems existing in the use of OAs are the delivery of OAs to cancer sites, the difficulty of transducing cancer cells that are deficient or poorly expressing adenovirus primary receptors, the specific and efficient replication in cancer cells, and the stimulation of immune responses. A multitude of basic research studies and clinical trials also focus on the efficacy and safety of OAs by overcoming these obstacles. Note that in 2015 Talimogene laherparepvec (T-VEC), an engineered herpes simplex virus type 1 (HSV-1), was approved by the by the U.S. Food and Drug Administration (FDA) and the European Medicine Agency (EMA). T-VEC, which is applied for the treatment of unresectable melanoma, represents the first OV approved by these agencies, giving people hope for using oncolytic viruses to treat cancers.

In comparison with other types of OVs, OAs can be produced at high final vector concentrations of up to 10^13^ plaque-forming units. In addition, the relatively small genome of around 36 kb, and established techniques enabling genetic genome modification, allow the insertion of foreign genes. As another advantage, the adenovirus genome displays low integration frequencies into host chromosomes, which, in contrast to other integrating vector systems, lowers the risk of inducing genotoxicity in transduced cells. However, OAs are more toxic than other OVs due to their higher immunogenicity and the relatively weaker cell killing effects require higher virus dosages to achieve a comparable oncolytic effect as, for instance, observed for HSV-1-based OVs.

In this review, we discuss approaches to deliver OAs, strategies for achieving cancer-cell-specific replication of OAs and the combination of them with other cancer therapies. In the context of these antitumor strategies, described in the following chapters, we mention important clinical trials utilizing OAs. To provide an overview of these studies, clinical trials based on OAs that are in the recruitment or completion state are summarized in Table 2. This includes information about the vector used, the outcome of completed clinical trials, the treated disease and the administration route. The majority of OAs are based on Ad type 5 (Ad5), which were broadly explored in vitro and in vivo to combat cancer. Here, we first discuss strategies which were utilized to arm and deliver these Ad5-based OAs, and how to combine these with other cancer therapies. However, besides Ad5, many other Ad types were identified (>100) with diverse features related to their tropism, receptor usage, cellular uptake, seroprevalence in the human population, immune responses and replication efficiencies in different cell lines. For these reasons, these alternative Ad types may provide a completely new resource to design novel oncolytic agents. Therefore, in the following chapters we provide a state-of-art overview to explore the complete natural diversity of human Ads as OAs. We briefly summarize molecular methods to convert alternative Ad types into OAs and we mention studies which screened other Ad types for cancer therapy. Furthermore, we introduce a cloned human Ad library consisting of numerous Ad types derived from the different species. Finally, we provide an outlook on the future of these alternative OAs and how the knowledge gained from studies using Ad5-based OAs can be used for the development of novel OAs with potentially improved features. 

## 2. Improving Delivery to Cancer Sites

**Figure 1:**
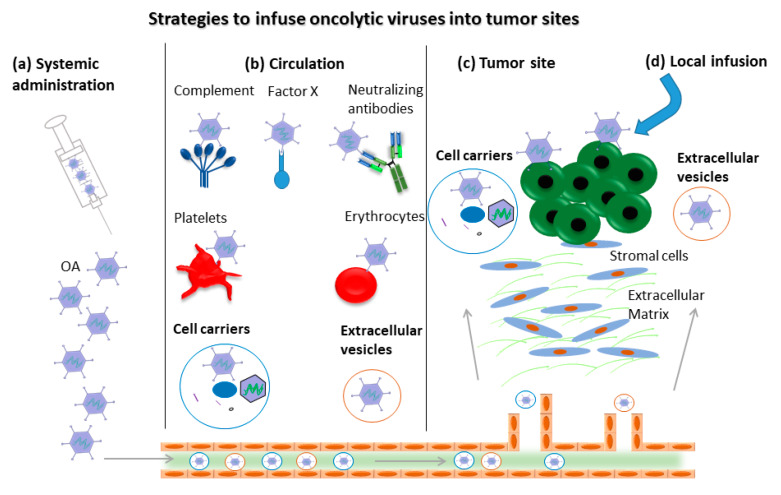
Strategies to overcome potential barriers in delivering oncolytic adenoviruses (OAs) into tumor sites. For systemic administration (**a**) such as intravenous injection, the complement, neutralizing antibodies, platelets and erythrocytes present in blood circulation bind to OAs (**b**) and block the migration of OAs into tumor sites. Cell carriers and extracellular-vesicles-delivered OAs escape the above-mentioned barriers to some extent before the OAs reach the tumor site (**c**). Local infusion represents an alternative strategy to avoid the interaction with blood components (**d**).

OVs were shown to selectively replicate in and kill cancer cells and provide a relatively safe strategy to facilitate the development of alternative cancer treatment options. However, AdVs can bind to neutralizing antibodies [20,21], and complement components [22], platelets [23] and erythrocytes (Figure 1) [24]. Consequently, systemically administrated OVs are rapidly cleared from the circulation and only a few OVs reach the cancer sites, potentially representing major hurdles for successful therapy. To overcome these obstacles, attempts to deliver OVs into cancer sites are on the agenda. Two strategies for carrying OVs with cell carriers were explored. The concept of OVs bound to the surface of the cell carrier has been used for a long time [25,26]. However, the binding of cell-surface-bound OVs hinders the delivery of OVs into cancer sites in the presence of pre-existing neutralizing antibodies and complement components in the serum. An alternative concept, may be to package the virus inside of the cell, which can then function as carrier, potentially improving the delivery effect.

To improve the cancer-specific recruitment, OAs were delivered by mesenchymal stem cells (MSC) [27,28]. The Ad5-based OA effectively transduces MSCs and replicated efficiently in these cell carriers. The OA loaded into MSCs could lyse hepatocellular carcinoma (HCC) cells in vitro under both normoxic and hypoxic conditions. In addition, the systemic administration of OA-delivered MSCs led to a high level of virion accumulation in the tumor and attenuate the hepatic localization of the vector. Finally, the MSC-delivered OA inhibited cancer growth and decreased hepatotoxicity. Note that MSC-mediated delivery of OAs based on Ad5 including ICOVIR-5 (NCT01844661) and DNX-2401 (NCT03896568) is being evaluated in clinical trials.

Neural stem cells (NSC) also showed promising potential in delivering Ad5-based oncolytic CRAd-Survivin-pk7 in treating brain cancer, especially glioblastoma multiforme (GBM) [29,30,31,32,33,34,35]. Note that NSC was more efficient than MSC in the delivery of OAs to tumor sites and that NSC-delivered CRAd-Survivin-pk7 was also applied in a clinical trial (NCT03072134).

Carrier cells derived from the immune system were also employed to deliver OVs into cancer sites. Immune cells such as dendritic cells [26,36], macrophages [37], peripheral blood mononuclear cells [38], lymphokine-activated killer cells [36], cytokine-induced killer (CIK) cells [39,40] and myeloid-derived suppressor cells (MDSCs) [41,42] were shown to function as cell carriers (reviewed in [43]). However, the performed studies revealed that the transduction of AdVs into immune cells is relatively poor, which represents a hurdle for the successful application of this approach in the clinic. As the only cell line product that has been infused into patients with advanced cancer with clinical benefit and minimal side effects (NCT00900809 and NCT00990717) [44,45], the natural killer cell-derived cell line NK-92 showed promise for carrying OVs. However, it remains to be shown whether this cell line can be applied as a carrier to deliver OAs. 

Cell-carrier-mediated systemic delivery of OA represents a promising strategy for improving anticancer efficacy and safety profiles. For the use of this therapy in the clinic, one issue should be taken into account, which is the arrival of cell carriers at the tumor site before the OA from the cell carriers is released. Therefore, the experimental setup should be planned carefully to guarantee that the cell carrier permits the virus to only replicate during the carrying process and then lyse the cell carrier to release the virus at the tumor site. 

In addition, cancer-cell-derived extracellular vesicles could also be purified and employed as OA delivery vehicles [46,47]. OAs were encapsulated inside extracellular vesicles (EV) and administered intravenously, resulting in cancer-selective delivery. However, no cancer tropism was observed with intraperitoneal administration. Furthermore, the EV-mediated delivery does not alter the immunomodulatory properties of the OAs. Figure 1 provides an overview of the strategies for delivering OAs into cancer sites.

Certainly, approaches based on the direct intratumoral injection (IT) of OAs omits the interaction with blood components such as binding to neutralizing antibodies, complements and blood cells. The administration of LOAd703 (NCT04123470, NCT02705196, NCT03225989), ONCOS-102 (NCT03514836, NCT01598129), VCN-01(NCT02045589), DNX-2401(NCT03178032, NCT02197169, NCT00805376, NCT01956734), and OBP-301 (NCT02293850, NCT03172819, NCT03213054, NCT03921021) through IT have been started or will be included in clinical trials (Table 2).

## 3. Enhancing Cancer-Specific Replication

There is a risk that OAs can infect both cancer cells and normal cells, representing a threat to normal tissues. To guarantee the safety of OAs, the specific replication in and killing of cancer cells is necessary. To date, different strategies have been utilized to improve the cancer-specific replication of OAs, according to the characteristics of the tumor type. 

Since neuroendocrine cancer cells secreting chromogranin A (CgA) have an activated gene regulatory element, Yu et al. employed the CgA promoter to control E1A expression for specific OA replication in neuroendocrine cancer cells [48]. Neuroendocrine cancer-bearing mice treated with OAs had a prolonged survival compared with placebo-treated mice [48]. Gastroenteropancreatic neuroendocrine (NET) cancers and lung NET cancers often cause liver metastases [49]. In order to reduce virus replication in normal hepatocytes, the target sequence for the liver-specific microRNA miR122 was incorporated in the 3’untranslated region of the E1A gene. Leaky mRNA expression of the oncolytic adenovirus was efficiently silenced by miR122 both in human hepatocytes in culture and in mouse liver in vivo [50]. To enhance the transduction of cancer cells with low CAR expression, a cell-penetrating peptide, the protein transduction domain (PTD) from the Trans-Activator of Transcription (Tat) protein of human immunodeficiency virus (HIV)-1, was inserted in the hypervariable region (HVR)-5 of the OA hexon protein, resulting in CAR-independent and optimized transduction of cancer cells [51]. Ad5-based AdVince combined the three targeting strategies (CgA promoter, miR122 target sequences, and PTD) and specifically killed cancer cells for the treatment of liver metastases from neuroendocrine cancer. The preclinical ex vivo evaluation of AdVince on human gastrointestinal NET cells isolated from liver metastases and human hepatocytes as well as human blood showed that AdVince selectively replicated in and killed NET cells. In addition, the toxicity of AdVince in hepatocytes was mild and AdVince induced negligible inflammation responses in human blood [52]. Safety, efficacy and immunological response are currently evaluated in Phase I/IIa trials in patients with metastatic NETs (NCT02749331).

Telomeres, representing repetitive nucleotide sequences at each end of the chromosomes, play a pivotal role in retaining genome stability [53,54,55]. The human telomerase reverse transcriptase (hTERT) is necessary for the human telomerase activity and its promoter triggers hTERT gene expression selectively in cancer cells and is silent in most normal cells [56]. Ad-hTERT is an *E1*-deleted and replication-defective OA and, therefore, the hTERT promoter can control the expression of E1 in telomerase-positive cancer cells, leading to selective replication in cancer cells. Currently, Ad-hTERT is an attractive OA and showed promising cancer-specific lysis [57,58,59]. Furthermore, the hTERT-controlled OBP-301 is in clinical trial to treat different cancers (NCT02293850, NCT03172819, NCT03213054, NCT03921021).

Survivin expression in gliomas is related with poor prognosis, higher recurrence, and resistance to chemo- and radiotherapy. For the Ad5-based OA CRAd-Survivin-pk7, the native E1 promoter was deleted to avoid viral replication in normal cells, and the human survivin promoter, termed CRAd-S, was inserted to drive E1 expression, resulting in the gliomas-specific replication (NCT03072134) [35].

## 4. Improving Anti-Tumor Effects through Combination of Currently Available Therapies

Different types of anti-tumor agents kill cancer cells and inhibit cancer growth through diverse mechanisms, but the application of a single agent in affected patients often shows poor efficacy. However, the combination therapy targeting different attack points in cancer progression revealed a mutual improvement compared to the single-agent concept, resulting in promising synergistic effects and benefits for the patient. 

### 4.1. Oncolytic Viruses in Combination with Surgery, Chemo- and Radiotherapy

The earliest strategy to combat cancer diseases is surgery to remove the tumor and metastasis. However, in concert combination with chemo- and radiotherapy and other advances in modern medicine, cancer treatment has advanced gradually in recent years. There are numerous examples demonstrating that these established treatment options result in synergistic effects in combination with OAs. The conditionally replicating adenovirus (CRAds), for instance, was genetically engineered by removing its E1B region and inserting a survivin promoter for cancer-specific replication. A combination of CRAd with cisplatin interfering with DNA replication resulted in improved cancer-killing efficacy in a synergistic manner. CRAd could transduce cisplatin-resistant lung cancer cells A549-DDPR [60]. Furthermore, the upregulation of CAR in breast cancer cells by cisplatin-enhanced, cancer-specific transduction of cancer cells with OA [61]. This indicated that the upregulation of oncolytic virus receptors on the cancer cell surface could be a selective vulnerability of chemotherapy-resistant cancers. The screening of multiple chemotherapy-induced OA receptors on cancer cells might provide clues for enhancing the synergistic efficacy of combining chemotherapy and OA. 

The oncolytic adenovirus CGTG-102 (Ad5/3-D24-GMCSF) encodes GM-CSF for stimulating anti-cancer immunity [62] and it could selectively replicate in p16/Rb-defective cells, which are a feature of most cancers [63,64,65]. To explore new treatment options for sarcomas, CGTG-102 was combined with the first-line chemotherapies for soft tissue sarcomas (STS), doxorubicin and/or ifosfamide [66]. A synergistic effect of CGTG-102 and doxorubicin plus ifosfamide was observed in Syrian hamster STS cells in vitro and in vivo in immunocompetent Syrian hamsters, which was associated with enhanced adenovirus replication. In addition, a phase II clinical trial with a phase Ib safety lead-in cohort of ONCOS-102 and cyclophosphamide in patients with unresectable malignant pleural mesothelioma is active (NCT02879669), and their combination for treating different solid tumors has been finished in another phase I clinical trial (NCT01598129). Gemcitabine is a synthetic pyrimidine nucleoside prodrug and has been widely used to treat a broad spectrum of cancers. To improve treatment efficacy, Gemcitabine was combined with LOAd703 (NCT03225989) and VCN-01(NCT02045589) to treat diverse cancers, especially pancreatic cancer. In addition, Temozolomide is an alkylating agent and a first-line treatment for glioblastoma multiforme. The phase I clinical trial combining DNX-2401 with Temozolomide for the treatment of glioblastoma multiforme has been completed (NCT01956734). 

It is well known that p53 is a central regulator of cellular damage response [67]. Diverse cellular stress could cause the accumulation of p53 protein in cells, which induces cell-cycle arrest and prevents the transformation of normal cells [68,69,70]. However, the *p53* gene is mutated in 60–80% of cancers and mutated p53 clearly contributes to the progression of human cancers [71]. Therefore, the restoration of p53 function is a potential alternative for treating cancers, and it was found that the gain of function (GOF) of a *p53* gene mutation in the transcriptional activation domain 2 (TAD2) suppressed cancer progression [72,73]. Based on this observation, the wild type *p53* gene and Ad5 were combined for the production of the recombinant adenovirus Gendicine [74,75]. Gendicine was approved by the China Food and Drug Administration (CFDA) in 2003, as a first-in-class gene therapy product to treat head and neck cancers [76].

Radiotherapy represents a conventional therapeutic option for cancers, and the improvement in efficacy and the decrease in toxicity are often achieved through combination with radiosensitizers [77]. However, radiosensitizers used for cancers represent cytotoxic chemotherapies, like cetuximab in radical head and neck radiotherapy [78]. Ads have inhibited the cellular DNA damage response to prevent the viral genome from being recognized by the cell, allowing successful viral replication [79,80,81]. This property of adenovirus indicates that the OAs in combination with radiotherapy might enhance radiation-induced cancer cell damage. Currently, the combination of OBP-301 and radiotherapy for the treatment of hepatocellular carcinoma in phase I clinical trial is active (NCT02293850).

Due to the radiosensitization by the CD/5-FC and HSV-1 TK/GCV enzyme/prodrug systems in addition to the chemotherapeutic effect [82,83,84,85,86,87,88], the efficacy of this oncolytic agent in combination with radiotherapy was evaluated in a clinical trial [89]. Ad5-yCD/mutTK(SR39)rep-ADP (Ad5-DS) was first introduced for the treatment of prostate cancer [90], and it is armed with “double suicidal genes”: Yeast cytosine deaminase (yCD) and herpes simplex virus 1 thymidine kinase (HSV-1 TK). Both genes were inserted into the E1 domain of adenovirus [90,91,92]. In a preclinical model of pancreatic cancer, Ad5-DS was injected into cancers, and the separately administered prodrugs of 5-fluorocytosine (5-FC) and valganciclovir (vGCV) are converted into their respective metabolites, 5-fluorouracil (5-FU) and valganciclovir-5-monophosphate (vGCV-MP) by yCD and HSV-1 TK genes, respectively [91]. In addition, the phase 2 clinical trial combining Ad5-yCD/mutTKSR39rep-ADP and radiotherapy has been completed (NCT00583492). 

CG7870 is a prostate-cancer-specific, replication-competent adenoviral vector. In this vector, the expression of the E1A and E1B genes are under the control of the rat probasin promoter and the human-prostate-specific antigen (PSA) promoter, respectively [93]. In established subcutaneous LNCaP xenografts in nude mice, CG7870 in combination with radiotherapy surpassed single treatment in decreasing the serum level of PSA and enhancing necrosis and the number of apoptotic cancer cells [94]. Furthermore, lower doses of OAs were used in combination therapy. Therefore, the combination therapy increased anticancer efficacy with no additional side effects if compared to a single treatment. In addition, Toth and colleagues studied Ad5-based vectors that overexpress an Ad5 protein named Adenovirus Death Protein (ADP, also named E3-11.6 K protein) in A549 lung cancer cells in combination with radiotherapy [95]. In this study, radiation increased the oncolytic activity of Ad5 and the combination led to improved effects in suppressing the growth of A549 lung adenocarcinoma xenografts in nude mice compared to a single treatment. The synergistic efficacy was also observed with different adenoviral vectors in the context of ovarian cancer cells [96] and glioma xenografts [97]. Furthermore, the minimal activity of OAs in normal cells displayed a selective radiosensitization effect in cancer cells [96,98].

### 4.2. Oncolytic Viruses in Combination with Immunotherapy

**Figure 2:**
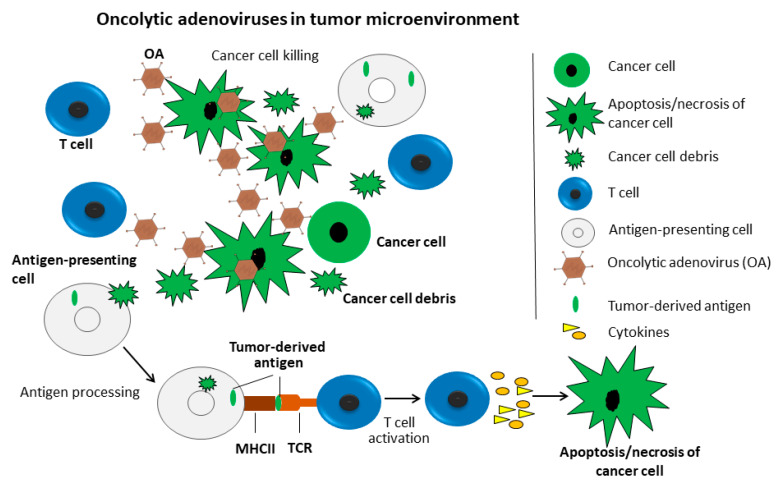
Oncolytic adenoviruses (OAs) in the tumor microenvironment. The OAs inhibit cancer growth through direct killing of cancer cells (green) and the stimulation of immunity in the tumor microenvironment. OAs replicate in and kill cancer cells, resulting cell debris, which are engulfed by macrophages (grey). Macrophages process cell debris-derived antigens and present them to T cells (blue). T cells are then activated and release cytokines, which induce apoptosis or necrosis of cancer cells.

The knowledge about the underlying molecular mechanism of OVs resulting in tumor killing experienced a complicated process. Initially, OVs were thought to inhibit cancer progression through direct replication in cancer cells, and subsequently kill them. There is accumulating evidence supporting the idea that OVs likely function through stimulating an immune response in the cancer microenvironment, which is further supported by the observation that OVs are inefficient in immune-deficient cancer models (Figure 2). The adenovirus virion induces innate immunity that is then translated into adaptive responses, and this is the theoretical basis of AdVs as a cancer vaccine [99]. Furthermore, the OA-replication produces an endogenous danger-signaling molecule in infected cancer cells that, in turn, stimulates dendritic cells (DCs), resulting in specific activity against cancer cells [100]. In particular, the specific immunity against cancer cells might be favorable for the long-term survival of treated cancer patients [101,102,103]. AdV replication inside tumor bulks is enhanced by the depletion of T cells, however, this also abrogates the efficacy of OAs-mediated anti-tumor efficacy, indicating the critical role of T cells in the effect of OAs [104]. 

Therefore, there is an increasing number of studies focusing on combining OVs with immune therapy as, for instance, the construction of OV-encoding cytokines. Note that approaches involving the combination of OA with granulocyte-macrophage, colony-stimulating factor (GM-CSF) and interleukin (IL)-12 were reviewed in detail elsewhere [105], and therefore this is not the focus of the present review. 

Cluster of differentiation 40 (CD40) has gained much interest as an immunostimulatory molecule in a variety of solid cancers [106,107,108,109,110,111], but the broad expression of CD40 in multiple cell types limited its cancer-specific application [109,110,111,112,113]. Therefore, the cancer cell membrane-bound CD40L for the local stimulation of a cancer-specific CD40 signaling is necessary to improve safety. Human 4-1BB ligand (4-1BBL) is documented to enhance the proliferation, activation, and survival of T cells [114,115,116], and could also expand natural killer (NK) cells [117,118]. To stimulate the anti-cancer immune response, the E3 region in the oncolytic adenovirus LOAd703 was replaced with the sequence encoding a designed trimerized membrane-bound isoleucine zipper (TMZ) TMZ-CD40L [119] and 4-1BBL under the control of a cytomegalovirus (CMV) promoter [120,121]. LOAd703 represents an Ad5-based chimeric oncolytic adenovirus containing an Ad5 fiber tail and shaft, and Ad35 knob [122,123]. Moreover, the palindromic E2F-binding site was inserted into the endogenous E1A promoter to improve the systemic toxicity profile and to increase the cytotoxicity to cancer cells [124]. In in vivo pancreatic cancer xenograft models, LOAd703 efficiently reduced established cancers, and its combination with gemcitabine further enhanced the efficacy. Currently, two Phase I/II trials that intervene with LOAd703 are in the recruitment process. The Phase I/II trial evaluates LOAd703 in patients with diverse types of cancers including pancreatic, biliary, colorectal and ovarian cancers, together with chemotherapy or using gemcitabine immune-conditioning (NCT03225989). 

Programmed cell death protein 1 (PD-1) is an immune regulating molecule that is expressed on T cells and inhibits T cells’ activation. In cancer patients, sufficient expression of PD-1 on T cells inhibits the killing of cancer cells by T cells [125]. Furthermore, the blockade of PD-1 and PD-1 ligand (PDL-1) in cancer therapy has presented promising durable anti-tumor responses and long-term remissions in patients with different cancers [126,127]. The humanized antibody pembrolizumab targeting PD-1 is combined with OBP-301 (NCT03172819) for the treatment of prostate cancer and other solid tumors. In one clinical trial, LOAd703-mediated oncolytic viral therapy for pancreatic cancer was evaluated, in which the OA-based therapy was combined with standard of care treatment including gemcitabine and nab-paclitaxel with or without the anti-PD-L1 antibody atezolizumab (NCT02705196). In this study, blood and biopsy samples from patients will be collected for analyzing the presence of LOAd703, atezolizumab cancer markers, and immunology markers. In another clinical trial (NCT04123470), the combination of LOAd703 and atezolizumab is explored for the treatment of melanoma. In addition, ONCOS-102 (NCT02963831) and VCN-01 (NCT03799744) are combine with the anti-PD-L1 antibody Durvalumab for treating peritoneal malignancies and head and neck neoplasms, respectively. 

DCs are potent antigen-presenting cells and are very potent in the stimulation of T cell-mediated immune responses [128]. The overexpression of wild-type or mutant p53 protein exists in approximately 50% of cancers. Nikitina and colleagues transduced DCs with AdVs coding wild-type p53 (Ad-p53), then used the processed DCs to stimulate T cells from HLA-A2-positive cancer patients. CD8^+^ T-cell-mediated cytotoxicity was detected against HLA-A2-positive cancer cells with the accumulation of p53, but not against HLA-A2-positive cancer cells with undetectable levels of p53 or against HLA-A2-negative cancer cells. This finding indicated that DCs transduced with p53 induced specific anti-cancer immune responses and that this combination therapy offers a promising approach [129]. In a phase I/II clinical trial, the combination of ONCOS-102 with dendritic cells (DCs) pulsed with killed LNCaP prostate cancer cells (DCVAC/PCa) for male patients with metastatic castration-resistant prostate cancer is in recruitment (NCT03514836, NCT02963831, NCT03003676). 

## 5. Potential of Exploring the Complete Adenovirus Spectrum in Treating Cancers

To achieve efficient adenovirus-based therapy for cancer, the very first factor to consider is cellular entry of the respective virus, which is dependent on the expression levels of the primary receptors on the targeted cancer cells. Previous studies revealed that the CAR, which is the functional receptor for Ad5, is absent in a variety of primary cancers [130,131,132,133]. Therefore, the therapeutic efficacy of Ad5-based vectors critically demands the expression of CAR on the targeted cancer cells. Hence, to improve therapeutic efficacy, one promising approach is to explore the broad spectrum of adenovirus types. To date, more than 100 human adenovirus types and more than 200 animal-derived Ads were identified. These AdVs display type- and species-dependent biodistribution patterns due to their diverse cellular receptor usage. Besides CAR, alternative receptors were identified. Most species B and some species D human AdVs have been confirmed to use a ubiquitously expressed membrane protein CD46 as primary cellular entry receptor [6,134]. While DSG2 is used by Ad3, Ad7, Ad11, and Ad14 as a major entry receptor, sialic acid (SA) is the primary receptor for some species D viruses and species G virus Ad52 [135,136,137]. There is only limited information about the biological characteristics for many of these 103 human adenovirus types. However, it can assumed that they differ with respect to their mechanisms for cellular uptake related to receptor usage, as described above, and intracellular trafficking, seroprevalence in the human population, immune responses and replication efficiencies in different cell lines. To take advantage of this large resource of naturally occurring Ads, there are two approaches to pursue. Either chimeric viruses are generated combining different characteristics of two different Ad types, or a complete serotype switch is conducted. Note that the first approach was predominantly undertaken for Ad5-based vectors containing capsid components of other Ad types with the goal of changing the tropism of these vectors. Both approaches are discussed in the following two chapters. 

### 5.1. Construction and Evaluation of Chimeric Oncolytic Adenoviruses

To expand the tropism of AdVs, engineered chimeric OAs containing capsids and fibers from different adenoviruses were introduced. In most cases, the chimeric AdVs demonstrated higher transduction efficiency and, subsequently, more potent tumor cell killing. Here, we discuss OAs, which are based on the commonly applied Ad5 containing modified fiber proteins derived from other Ad types (Ad11, 35, 3, and 40). 

The infection of Ad5 into cells mainly depends on the CAR present on target cells. However, down-regulated CAR expression levels in cancer cells impedes Ad5-mediated gene transfer. Ad11 and Ad35 belong to the subtype B group and use CD46 as their cellular receptor, which is widely expressed on diverse tumor cell types [138,139]. To extend Ad5 tropism, the Ad5-based chimeric Ad5/11 and Ad5/35 vectors were constructed, in which the Ad5 fiber was substituted with that of types 11 or 35, respectively. The in vitro models showed that Ad5/35 infected pancreatic and breast cancer cells more efficiently than Ad5 and Ad5/11 [140]. Moreover, the Ad5/35-based oncolytic vectors showed more promising efficacy than the Ad5-based vectors for the treatment of experimental glioblastoma [141]. Furthermore, CD46 expression levels on colorectal cancer cells were positively correlated with Ad5/35-mediated GFP fluorescence and its oncolytic effect [141]. In addition, the Ad5/35 vector also delivered the anti-HER2 receptor into macrophages to obtain the chimeric antigen receptor engineered macrophages. The Ad5/35-transduced engineered macrophages showed M1 polarization that plays critical roles in anti-tumor response [142]. In clinical trials, the knob domain of Ad5 was substituted with the corresponding domain of Ad3 in Oncos-102 (NCT03003676, NCT03514836, NCT02963831, NCT01598129), the fiber domain of Ad5 was substituted with the corresponding domain of Ad35 in LoAd703 (NCT04123470, NCT02705196, NCT03225989) and a partial E2B of Ad11p was replaced by the Ad3 E2B in Colo-Ad1(NCT02053220). 

The research performed on gliomas compared the oncolytic effect of Ad5-based OAs with different fiber modifications. These OAs contains an RGD motif incorporated into the HI loop of the knob protein (CRAd-S-RGD), a substitution of the Ad5 fiber knob for the Ad3 knob (CRAd-S-5/3), or a polylysine modification of the fiber knob (CRAd-S-pk7). The research indicated that CRAd-S-pk7 provided promising results referring to viral replication and tumor oncolysis in glioma cell lines. Moreover, experiments performed in a xenograft model showed that CRAd-S-pk7 could inhibit tumor growth and prolong survival significantly [35]. 

Ad3 belongs to species B Ads. The Ad5-based oncolytic Ad5/3-Δ24 vector was constructed to overcome CAR downregulation in cancer cells, which then allowed the effective transduction of CAR-negative cancer cells [143]. In addition, the transduction-enhanced Ad5/3-D24-tras vector could encode trastuzumab antibody heavy- and light-chain, and the transduced cancer cells assembled full-length functional antibody [144]. The Ad5/3-D24-tras showed improved antitumor efficacy if compared to Ad5/3-Δ24 or trastuzumab, and it also induced dendritic cell activation and natural killer cell accumulation in tumor-draining lymph nodes.

The enteric Ads Ad40 and Ad41 belong to the species F and both virions possess two fibers of different lengths and characteristics [145]. The long fiber can attach to the CAR receptor, but the short fiber lacks CAR-binding properties [146]. The lack of CAR binding of the short fibers derived from Ad40 and Ad41 provides the potential to change the tropism of chimeric AdVs. The Ad5-based chimeric Ad5/40S contains the short fiber of Ad40 (F40S) and it presented dramatically reduced transduction efficiencies in the liver and spleen if directly compared with Ad5 vectors [147]. However, the incorporation of a 7-lysine-residue motif at the C-terminal end of the Ad40 short fiber in Ad5/40S recovered the transduction efficiencies. The chimeric Ad5SHORT that carries the Ad5 genome and Ad41 short fiber exhibited decreased transduction of human intestinal epithelial cells [148]. Therefore, Ad5/40S and Ad5SHORT are good candidates to employ bispecific conjugates to link the viral capsid and different target cancer cells, while avoiding the transduction of cells expressing the CAR receptor [149].

### 5.2. Using the Natural Diversity of Human Adenoviruses and Converting Them into Oncolytic Viruses

To overcome the shortage observed in Ad5-based cancer therapy, a broad spectrum of wild type (wt) AdVs have been studied in primary cancer cells. Hoffmann and colleagues evaluated 20 wt-human AdVs in two individual cancer models. In both studies, the cellular entry of adenovirus was applied as a decisive parameter for infectivity. In the soft tissue sarcoma model, several Ad types demonstrated a higher internalization efficiency than Ad5, such as Ad types 35, 3, 7, 11, 9 and 22 [150]. This result is in accordance with the fact that these Ad types can use alternative cellular receptors to achieve CAR-independent infection. However, cellular entry is only the very first step of cancer therapy, leaving the question of how other factors like transgene expression levels and virus replication rates influence OA efficacy. To test whether the Ad types displaying the highest internalization rates can also result in enhanced cancer therapy, this strategy was explored for Ad35. Besides a parental conditionally replication-competent adenoviral vector, Ad5d24.Ki-COX, the OA Ad5/F35d24.Ki-COX was generated. The latter vector was based on the identical molecular design, except that the original Ad5 fiber was replaced by the Ad35 shaft and knob domain. As expected, the vector containing the Ad35 fiber showed significantly higher oncolytic efficiency than the parental Ad5 vector in vitro and in vivo in a xenograft model. In a second study, performed by the same research group, this panel of wt-AdVs was evaluated in malignant melanoma cells [151]. Interestingly, the authors also studied the expression levels of the two major adenovirus receptors CAR and CD46 in primary melanoma cells and the immunohistochemical stainings of primary cutaneous of melanoma lesions from five patients were negative for CAR and positive CD46 expression. Note that the in situ immunohistochemistry data could be confirmed by flow cytometric analysis of the short-term cultures prepared from these melanoma lesions. 

Similar to the first study, some Ad types (35, 38, 3, 49, 21, 34 and 7) demonstrated a higher internalization efficiency than Ad5 in melanoma cells [151]. Meanwhile, the enhanced oncolytic potential of Ad35 was proven in both in vitro and in vivo models. Later, in 2011, Chen and colleagues compared 13 alternative Ads representing species B, C, D, and E to Ad5 regarding their oncolytic potential [152]. Experiments were performed in both immunodeficient mice and immunocompetent hamster models, and it was found that Ad6-based oncolytic therapies for the treatment of breast, ovarian, kidney, and liver cancers were most efficient. Another study from the same group tested 15 Ad types in various cell lines and primary patient B-cell cancers [153]. The infection, replication and cancer cell killing rates were applied to identify candidates for novel OAs. Species D AdVs were discovered to have the highest potential as oncolytic agents against B-cell cancers, and Ad26 and Ad45 can notably delay lymphoma growth in xenograft in vivo models with a single treatment.

### 5.3. Converting Alternative Serotypes into Oncolytic Adenoviruses

A previous study constructed an Ad5-based library with recombinant viruses carrying fibers from other human Ads [133]. The authors demonstrate that these vectors display a divers topism and show that these fiber-chimeric vectors have potential for therapeutic applications. However, it can be speculated that studies based on wt-AdVs may lead to a better understanding of the cancer treatment potential of individual Ad types. However, for quite some time, the construction of engineered Ad genomes was hindered by available technologies. These were needed to gain genetic access to the complete spectrum of human Ads. To meet the increasing number of adenovirus types identified, a high throughput (HTP) strategy is mandatory. Our research group recently developed a method for the HTP direct cloning of Ad genomes from purified particles. This strategy is based on genomic DNA preparations from infected cells and cloning gof complete Ad genomes utilizing RecET-mediated linear–linear homologous recombination (LLHR), followed by rapid adenoviral genome modification and tagging by λ Red-mediated linear–circular homologous recombination (LCHR) [154,155,156]. Thereby, we established a novel adenovirus library of unprecedented diversity, comprising 34 types of the seven Ad species known to infect humans and around 20 adenoviral vectors tagged with reporter genes [157]. Notably, the reporter-labeled adenovirus types are suitable for HTP approaches to identify Ads for disease-specific targeting. Transgene expression can be efficiently evaluated by measuring either GFP or luciferase expression levels in infected cells. Other technologies were also explored for cloning complete Ad genomes, including modular assembly [158,159,160]. 

**Figure 3:**
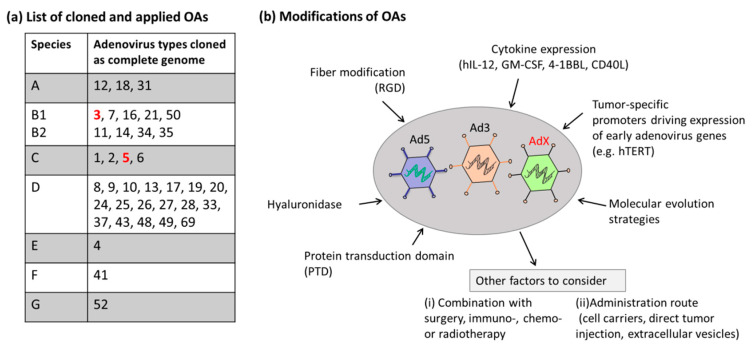
Cloned adenoviruses and modifications. (**a**) List of cloned wild-type adenoviruses which can be converted into tumor-specific OAs in the future. Adenovirus types marked in red (Ad5 and Ad3) were converted into OAs and also applied in the clinic. (**b**) To improve the efficacy of OAs and to arm novel OA candidates (AdX), diverse strategies were explored in clinical trials. Cytokine expression and release by OAs are able to stimulate immunity in the tumor microenvironment. Tumor-specific promoters, such as the hTERT promoter, guarantee tumor-specific replication of OAs and improve safety. The protein transduction domain (PTD) could penetrate cancer cells and enhance the transduction of OAs into cancer cells. Fiber modification such as the insertion of the tripeptide Arg-Gly-Asp (RGD) motif into the shaft of OAs, improves the binding of OAs to cancer cells. The enzyme hyaluronidase could be added as a transgene, which could degrade hyaluronic acid in the extracellular matrix and facilitate the spread of OAs through the tumor microenvironment. Furthermore, molecular evolution strategies can be applied. Other factors to consider are the administration route and the combination with conservative cancer therapies.

In our previous work, we applied this tagged Ad library to identify novel Ad candidates showing improved uptake into various immortalized cell lines from various tissues and investigated the oncolytic potency of these AdVs in osteosarcoma cells. We found that the transduction rates and oncolytic potential of the different Ad types are Ad-type-specific. However, this is only the first step in developing novels Ads for cancer therapy. As a further step, these cloned Ad genomes need to be converted into OAs by arming them with transgenes encoding for instance cytokines, and by using strategies rendering the replication of these Ads cancer cell specific as, for instance, tumor-specific promoters driving E1 expression (e.g., the hTERT promoter). As demonstrated for Ad5-based OAs, the oncolytic efficacy can also be combined with commonly applied cancer therapies such as immunotherapy, chemotherapy and radiotherapy. A list of cloned Ads, which are accessible for genetic modification and subsequent conversion into OAs, and the OAs which were already applied, are listed in Figure 3. Furthermore, this figure mentions strategies, which can be applied.

Besides OAs based on Ad5, the most prominent Ad candidate which was converted into OAs is based on Ad type 3 derived from species B viruses. The Ad3-based Ad3-hTERT-E1A vector was developed and showed hints of tumor killing in both tumor patients and animal models [161]. Moreover, a modified version of this OA was generated (Ad3-hTERT-CMV-hCD40L), which was armed with an expression cassette encoding the human immune-stimulatory molecule CD40L for modulation of the tumor microenvironment. The application of this OA resulted in improved function of dendritic cells and direct tumor lysis [162].

In another study, an Ad3/Ad11p chimeric oncolytic virus (ColoAd1) was generated by directed evolution [143], which represents another rational strategy to generate novel OAs. Further genetic analysis revealed that the major capsid proteins of this chimeric vector were derived from Ad11, attaching to CD46 that is widely expressed on tumor cell surfaces [138,139]. This replication- competent vector could bind CD46 and transduce many cell types with a higher efficiency than Ad5 [163]. Moreover, the seroprevalence of Ad11 in humans is very low and has no cross-reactivity with Ad5 [129]. ColoAd1 could amplify and lyse tumor cells efficiently and showed anti-tumor activities, which were superior to the clinically approved oncolytic viruses HAdV-C5, ONYX-015 [143]. 

It was demonstrated that the Ad3-based oncolytic adenovirus Ad3-hTERT-E1A induced less liver damage than Ad5 or Ad5/3 in mice cancer models [161,164]. Although in vitro oncolysis mediated by Ad3-hTERT-E1A showed less oncolytic efficacy than with Ad5 or Ad5/3, its in vivo potency is comparable to the other vectors. Furthermore, the anti-tumor efficacy of Ad3-hTERT-E1A was not decreased in the presence of neutralizing anti-Ad5 antibodies, although in the Ad3-hTERT-E1A treatment, T-cell activation against Ad3 hexon was found in Ad5 pretreated patients, but not in naive patients, indicating the crossreactivity between T-cell epitopes [165]. Possible efficacy was still seen in 73% of treated patients, and particularly promising results were obtained in breast cancer patients receiving concomitant trastuzumab. Therefore, Ad3-hTERT-E1A offers an attractive alternative for existing OAs due to its safety and efficacy.

Taken together, the broad spectrum of AdVs provides a unique opportunity to identify the most suitable candidate for individual cancer treatment. For the comparison of adenovirus types, multiple indicators should be applied, such as cellular entry, transgene expression, and virus replication efficiency.

## 6. Conclusions

Transduction into both quiescent and dividing cells, the large cargo capacity and a broad tropism render AdVs a widely explored vector system. Currently, AdVs provide a potential platform for cancer treatment, mainly through oncolysis and delivery of genes of interest. An increasing number of basic research studies, pre-clinical studies, and clinical trials have shown the feasibility and safety of AdVs in cancer treatments. Note that the majority of clinical trials are still in the phase I stage of clinical trials, evaluating the toxicity and safety of the novel treatment option. Moreover, many clinical trials were not completed yet, making it impossible to draw clear conclusions about the benefits for the patients. Therefore, it may be difficult to predict which approach, and especially which clinical trial, listed in Table 2 may result in the best clinical outcome for the patient. Certainly, there are various factors which need to be considered for the outcome and future optimization of OAs. Cancer complexity influences the efficacy of AdVs and poses many challenges to the utility of AdVs in cancer treatments. There are still various limitations to be overcome to enhance the efficacy of AdVs in cancer therapy with respect to different aspects. Targeted delivery, efficient transduction, robust replication, evasion from cancer suppression and the stimulation of strong anti-cancer responses need to be optimized for achieving efficient cancer treatments with AdVs. Moreover, the cancer heterogenicity also poses new challenges for OV therapy. The combination with other anti-cancer agents is able to target different cancer progression mechanisms, and the mutual effect could enhance single treatment options, resulting in synergistic effects. Moreover, the development of an Ad library paves the way towards identifying alternative Ad candidates, which can be converted into OAs. Besides these points, AdV-mediated gene delivery, cancer cell lysis and modulation of cancer microenvironment shows great promise in the inhibition of cancer progression. 

## Figures and Tables

**Table 1:** Types and features of human adenoviruses.

Species	Serotype	Identified Receptor(s) for Members of this Species	Tropism
**A**	12, 18, 31, 61	CAR	Cryptic (enteric, respiratory)
**B**	3, 7, 11, 14, 16, 21, 34, 35, 50, 55, 66, 68, 76–79	CD46, DSG2, CD80, CD86	Respiratory, renal, ocular
**C**	1, 2, 5, 6, 57, 89	CAR, VCAM-1, HSPG, MHC1-a2, SR	Respiratory, ocular, lymphoid, hepatic
**D**	8–10, 13, 15, 17, 19, 20, 22–30, 32, 33, 36–39, 42–49, 51, 53, 54, 56, 58–60, 62–65, 67, 69–75, 80–88, 90–103	SA, CD46, CAR	Ocular, enteric
**E**	4	CAR	Respiratory, ocular
**F**	40, 41	CAR	Enteric
**G**	52	CAR, SA	Enteric

Note 1: CAR, coxsackievirus- and adenovirus receptor. DSG2, desmoglein-2. MHC1-a2, major histocompatibility complex-a2. SA, sialic acid. SR, scavenger receptor. VCAM-1, vascular cell adhesion molecule-1. HSPG, heparin sulfate proteoglycans.

**Table 2:** Adenoviral vectors for cancer treatment in clinical trials.

OncAd	Modification	Transgene	Disease	Administration	Phase	Clinical Trial NO.	Clinical Trial State	Combination	Main Results of Completed Studies
Ad5-yCD/mutTKSR39rep-ADP	E1B55K-deleted (the 34183bp to 35673bp encoding 55 kDa protein in E1B region is deleted)	yCD/TK	Prostate Cancer	IPR	2	NCT00583492	Completed	Radiation	No serious virus-related side effects; meaningful reduction in positive biopsy results for 2 years [11]
Ad5-yCD/mutTKSR39rep-hIL12	E1B55K-deleted (Same as above)	yCD/TK, hIL-12	Metastatic pancreatic cancer	IV	1	NCT03281382	Recruiting		n/a
Ad.hIFN-beta	E1/partial- deleted, E3-deleted	IFN-α2b	Mesothelioma	IPL	1	NCT01119664	Completed	Pemetrexed /Cisplatin	No serious toxicities; no biologic parameters found correlating with reponses to the treatment; median overall survival time of 12.5 months [12]
AdVince	CgA-E1A (CHGA gene (CgA promoter)-driven E1A expression)	miR122 target sequences, PTD	Neuroendocrine tumors	IH	1, 2	NCT02749331	Recruiting		n/a
CG0070	E2F-E1A (E2F-1 promoter-drivenE1A expression)	GM-CSF	Bladder Cancer	IVE	2	NCT02365818	Completed		No serious adverse effects; an overall 47% (21/45) CR rate at 6 months for all patients and 50% for patients with CIS [13]
Colo-Ad1	Ad11p/Ad3 (Ad11p backbone with a large deletion in the E3-region, a small E4-domain (E4orf4) deleted, and a partial E2B substitution by the Ad3 E2B)		Colon, NSCLC, Bladder, Renal Cancer	IT, IV	1	NCT02053220	Completed		No treatment-associated serious adverse events; specific virus delivery in most tumor samples, high local CD8^+^ cell infiltration in 80% (8/10) of tumor samples [14]
CRAd-S-pk7	Survivin promoter-driven E1A expression	pk7 (polylysine)	Brain Cancer	NSC, IC	1	NCT03072134	Completed	Radiation, chemotherapy	Not reported
DNX-2401	E1A Δ24, RGD (a 24 bp deletion (bp 923-946; the Rb-binding domain) in the E1A gene and the insertion of an RGD integrin-binding motif (4C peptide: Cys-Asp-Cys-Arg-Gly-Asp-Cys-Phe-Cys) in the H1 loop of the Ad fiber)		Glioblastoma; gliosarcoma tumor	IT	2	NCT02798406	Active, not recruiting	Pembrolizumab	n/a
			Brain cancer	BM-hMSCs, IA	1	NCT03896568	Recruiting		n/a
			Brainstem glioma	IT	1	NCT03178032	Completed		Not reported
			Glioblastoma, Gliosarcoma	IT	1	NCT02197169	Completed	IFNγ	No serious virus-related adverse effects; poor tolerability of IFNγ; IFNγ did not provide additional benefit; 50% of patients with a baseline tumor diameter of ≤ 42 mm survived beyond 12 months
			Brain cancer	IT, CI	1	NCT00805376	Completed		No serious toxicities; active virus replication in tumor; median overall survival time of 9.5 months for single IT; median overall survival time of 13.0 months for permanently implanted catheter [15]
			Glioblastoma Multiforme	IT, IM	1	NCT01956734	Completed	Temozolomide	No severe virus-related toxicities; FGF2 as a prognostic biomarker of DNX-2401 treatment response [16]
ICOVIR-5	E2F-E1A Δ24, RGD (E2F-1 promoter-drivenE1A Δ24 expression, the insertion of an RGD integrin-binding motif (4C peptide) in adenoviral fiber)		Melanoma	IV	1	NCT01864759	Completed		Not reported
			Solid tumors	MSC, IV	1, 2	NCT01844661	Completed		No serious toxicity; adenovirus replication in 78% (7/9) patients; circulating CD8+T cells raised [17]
LOAd703	5/35, E1A-Δ24 (Substituting the fiber domain of Ad5 with the corresponding domain of Ad35, E1A-24 bp in Ad5 is deleted)	TMZ-CD40L and 4-1BBL	Malignant melanoma	IT	1, 2	NCT04123470	Recruiting	Atezolizumab	n/a
			Pancreatic cancer	IT	1, 2	NCT02705196	Recruiting	Atezolizumab, nab-paclitaxel	n/a
			Pancreatic, Ovarian, Biliary, Colorectal cancer	IT	1, 2	NCT03225989	Recruiting	Gemcitabine/Cisplatin, Gemcitabine/Oxaliplatin	n/a
OBP-301	hTERT promoter (hTERT promoter-driven E1A expression)		Hepatocellular carcinoma	IT	1	NCT02293850	Recruiting	Radiation	n/a
			Advanced solid tumor	IT	1	NCT03172819	Recruiting	Pembrolizumab	n/a
			Esophageal cancer	IT	1	NCT03213054	Recruiting		n/a
			Esophagogastric adenocarcinoma	IT	2	NCT03921021	Recruiting		n/a
ONCOS-102	Ad5/Ad3, E1A-Δ24 (Substituting the knob domain of Ad5 with the corresponding domain of Ad3, E1A-24 bp in Ad5 is deleted )	GM-CSF	Melanoma	IT	1	NCT03003676	Active, not recruiting	Cyclophosphamide, Pembrolizumab	n/a
			Prostate cancer	IT	1, 2	NCT03514836	Recruiting	DCVAC/PCa, Cyclophosphamide	n/a
			Peritoneal malignancies	IP	1, 2	NCT02963831	Recruiting	Durvalumab	n/a
			Solid tumour	IV, IT	1	NCT01598129	Completed	Cyclophosphamide	No dose limiting toxicity or maximum tolerated dose was identified; 40% (4/10) evaluable patients had disease control based on PET/CT scan at 3 months; a prominent infiltration of TILs to tumors was seen post-treatment in 92% (11 /12) patients; median overall survival was 9.3 months [18]
VCN-01	DM-1-E2F-E1A Δ24, RGD (insulate the E2F1 promoter using DM-1 insulators, E2F-E1A Δ24 and RGD as ICOVIR-5)	Hyaluronidase	Head and neck neoplasms	IV	1	NCT03799744	Recruiting	Durvalumab	n/a
			Pancreatic adenocarcinoma	IT	1	NCT02045589	Completed	Gemcitabine and Abraxane®	Not reported

Note 1: Clinical trial data are from ClinicalTrials.gov: Home [19]; Note 2: oncolytic adenoviruses (OAs) listed in alphabetical order in this table are Ad5-based vectors. If modifications were introduced into the vector, these are indicated in the “Modification” column; Note 3: Abbreviation: BM-hMSCs, bone-marrow-derived human mesenchymal stem cells delivery. CgA, chromogranin A. CI, cancer surrounding brain tissue injection. CIS, carcinoma-in-situ. CR, complete remission. DCVAC/PCa, dendritic cells (DCs) pulsed with killed LNCaP prostate cancer cells. EV, endovenous injection. FGF2, fibroblast growth factor 2. GM-CSF, granulocyte-macrophage colony-stimulating factor. hTERT, human telomerase reverse transcriptase. IA, intra-arterial injection. IC, intracerebral injection. IFNγ, interferon gamma. IH, intrahepatic artery infusion. IM, intramural injection. IP, intraperitonealy infusion. IPL, intrapleural infusion. IPR, intraprostatic infusion. IT, intratumoral injection. IV, intravenous injection. IVE, intravesical intervention. MSC, mesenchymal stem cell delivery. n/a, not applicable because results of this trial are not available. NSC, neural stem cell. NSCLC, non-small cell lung cancer. PTD, protein transduction domain. RGD, Arg-Gly-Asp amino acid sequence as target of αv integrins. TK, herpes simplex virus 1 thymidine kinase. TMZ, trimerized membrane-bound isoleucine zipper. yCD, yeast cytosine deaminase.
